# Metabolic Profiling of Primary Metabolites and Galantamine Biosynthesis in Wounded *Lycoris radiata* Callus

**DOI:** 10.3390/plants9111616

**Published:** 2020-11-20

**Authors:** Chang Ha Park, Ramaraj Sathasivam, Bao Van Nguyen, Seung-A Baek, Hyeon Ji Yeo, Ye Eun Park, Haeng Hoon Kim, Jae Kwang Kim, Sang Un Park

**Affiliations:** 1 Department of Crop Science, Chungnam National University, 99 Daehak-Ro, Yuseong-gu, Daejeon 34134, Korea; parkch804@gmail.com (C.H.P.); ramarajbiotech@gmail.com (R.S.); guswl7627@gmail.com (H.J.Y.); yeney1996@cnu.ac.kr (Y.E.P.); 2 Department of Smart Agriculture Systems, Chungnam National University, 99 Daehak-Ro, Yuseong-gu, Daejeon 34134, Korea; nguyenvanbao@tuaf.edu.vn; 3 Division of Life Sciences, College of Life Sciences and Bioengineering, Incheon National University, Yeonsugu, Incheon 22012, Korea; bsa1103@inu.ac.kr; 4 Department of Well-being Resources, Sunchon National University, Suncheon 57922, Korea; cryohkim@sunchon.ac.kr

**Keywords:** *Lycoris radiata*, wounding stress, metabolic profiling, galantamine biosynthesis

## Abstract

Plants are continuously exposed to abiotic and biotic factors that lead to wounding stress. Different plants exhibit diverse defense mechanisms through which various important metabolites are synthesized. Humans can exploit these mechanisms to improve the efficacy of existing drugs and to develop new ones. Most previous studies have focused on the effects of wounding stress on the different plant parts, such as leaves, stems, and roots. To date, however, no study has investigated the accumulation of primary and galantamine content following the exposure of a callus to wounding stress. Therefore, in the present study, we exposed *Lycoris radiata* calli to wounding stress and assessed the expression levels of several genes involved in metabolic pathways at various time points (0, 3, 6, 12, 24, 48, 72, and 96 h of exposure). Furthermore, we quantify the primary and galantamine content using gas chromatography–time-of-flight mass spectrometry and the high-performance liquid chromatography qRT-PCR analysis of eight galantamine pathway genes (*LrPAL-2*, *LrPAL-3*, *LrC4H-2*, *LrC3H*, *LrTYDC2*, *LrN4OMT*, *LrNNR*, and *LrCYP96T*) revealed that seven genes, except *LrN4OMT*, were significantly expressed following exposure to wounding stress. Galantamine contents of calli after 3, 6, 12, 24, 48, 72, and 96 h of exposure were respectively 2.5, 2.5, 3.5, 3.5, 5.0, 5.0, and 8.5 times higher than that after 0 h of exposure. Furthermore, a total of 48 hydrophilic metabolites were detected in the 0 h exposed callus and 96 h exposed callus using GC-TOFMS. In particular, a strong positive correlation between galantamine and initial precursors, such as phenylalanine and tyrosine, was observed.

## 1. Introduction

Alkaloids are a class of natural organic compounds that contain at least one nitrogen atom in their structure, typically insoluble in water and physiologically active. These compounds are mainly synthesized by various living organisms including bacteria, fungi, plants, and animals [1]. Under unfavorable conditions, such as biotic or abiotic stress, plants produce alkaloids. Owing to these properties, alkaloids are suitable candidates for drug discovery and development and have therefore garnered increasing attention of scientists [2]. Over recent years, various alkaloids were isolated and identified from members of the Amaryllidaceae and have been reported to exhibit a wide range of pharmaceutical and biological activities [3].

*Lycoris radiata* belongs to the subfamily Amaryllidoideae of Amaryllidaceae. It is native to China, Korea, and Nepal, but it was subsequently introduced to the United States and Japan, in addition to other regions of the world [4]. Extracts from the plant display biological activities, such as acetylcholinesterase (AChE) inhibitory, anticancer, anti-inflammatory, antimalarial, antimicrobial, and antihypertensive properties, as well as cytotoxicity and neuroprotective effects [5,6,7,8]. In previous studies, over 20 alkaloids with neuroprotective effects [5,6,9] were identified from *Lycoris radiata*. Among these, galantamine (GAL) is an important alkaloid, which serves as an AChE inhibitor and a drug for Alzheimer’s disease. GAL has been approved for clinical use in several countries [3,4]. However, owing to low GAL levels in natural sources and its growing value in chemotherapy, an increasing number of studies have focused on elucidating the key enzymes and genes involved in the GAL biosynthetic pathway and GAL production in plants [10].

Plant callus can attain artificial control to provide the optimal growth conditions for the differentiation and accumulation of various secondary metabolites. In addition, plant callus consists of entire genetic information of whole plants, they have the totipotency for the synthesis of various secondary metabolites [11,12]. Moreover, the growing of callus culture in flasks on shakers or in fermenters has the ability to convert to single-cell suspension cultures to produce desired commercially important secondary metabolites [13]. This technology is one of the most important methods for the biosynthesis of various secondary metabolites from the callus. In various medicinal plants, the callus suspension culture system has been recognized throughout the world but the wounded *L. radiata* callus and analysis of secondary metabolites have not yet been studied [14,15,16]. Previously, few studies have been carried out to analyze the secondary metabolite content in the bulb, leaf, and root of *L. radiata* [4]. However, it remains unclear whether these secondary metabolites are also present in *L. radiata* callus.

**Figure 1:**
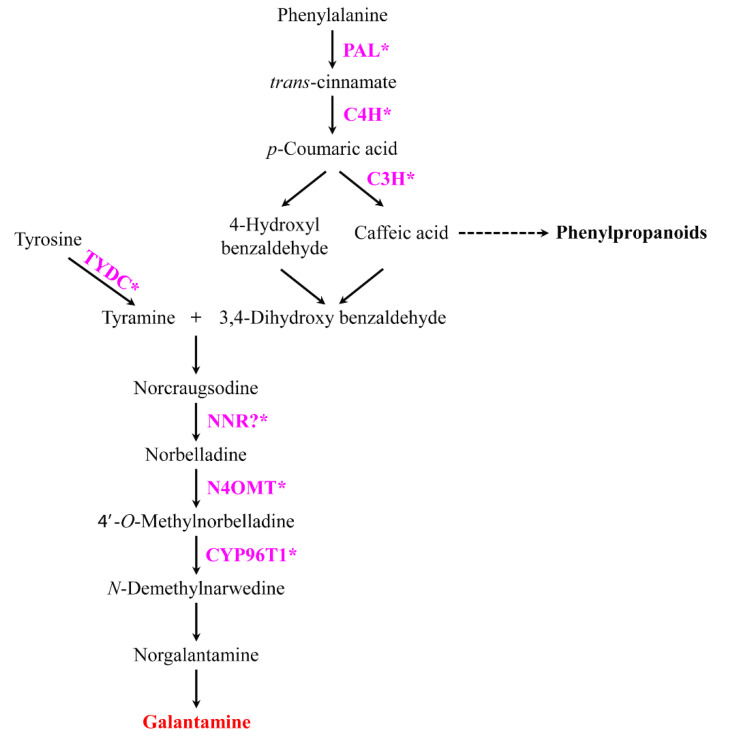
Proposed galantamine biosynthetic pathway in *Lycoris radiata*. Enzymes involved in each conversion reaction are indicated in pink. Asterisks indicate the genes used in expression analysis. Galantamine content measured using high-performance liquid chromatography is indicated in red. PAL, phenylalanine ammonia-lyase; C4H, trans-cinnamate 4-monooxygenase; C3H, *p*-coumarate 3-hydroxylase; TYDC, tyrosine decarboxylase; NNR, noroxomaritidine/norcraugsodine reductase; N4OMT, norbelladine 4′-*O*-methyltransferase, CYP96T1, noroxomaritidin synthase 1.

GAL is mainly synthesized in the leaf and bulbs of various plants such as *Galanthus elwesii* (0.045–0.082%), *Galanthus nivalis* (0.038–0.075%), *Galanthus woronowii* (0.040–0.078%), *Narcissus pseudonarcissus* (0.10–0.13%), *Leucojum aestivum* (0.13–0.14%), and *Lycoris radiata* (0.27–0.75 mg·g^−1^ dry weight (DW)) [4,17,18,19]. To date, however, the genes and enzymes involved in the biosynthesis of GAL in plants remain unclear compared to those in the biosynthesis of other secondary metabolites (carotenoids and phenylpropanoids). Phenylalanine and tyrosine are synthesized through the shikimate pathway, and phenylalanine is converted to trans-cinnamic acid (in a reaction catalyzed) by the enzyme phenylalanine ammonia-lyase (PAL) (Figure 1). Following this, the hydroxylation of trans-cinnamic acid into *p*-coumaric acid is catalyzed by the enzyme trans-cinnamate 4-hydroxylase (C4H) [20,21]. *p*-Coumaric acid is furtherly converted into two compounds namely 4-hydroxybenzaldehyde and caffeic acid, resulting in the production of 3,4-dihydroxybenzaldehyde [22]. In another branch of the pathway, tyrosine is catalyzed to tyramine by the tyrosine decarboxylase (TYDC) [23]. Through condensation and reduction, 3,4-dihydroxybenzaldehyde and tyramine converted to norbelladine by the enzyme noroxomaritidine/norcraugsodine reductase (NNR) [20]. However, the precise function of NNR remains unclear. Furthermore, through methylation, norbelladine is converted to the 4′-*O*-methylnorbelladine by norbelladine 4′-*O*-methyltransferase (N4OMT) [24]. Following this step, 4′-*O*-methylnorbelladine is converted to N-demethylnarwedine, which is subsequently converted to norgalantamine. Finally, norgalantamine is converted to GAL. However, the enzymes responsible for GAL synthesis have not been identified [4]. 

Various recent studies have reported that abiotic stress such as wounding, UV exposure, atmospheric changes, and exogenous phytohormone application, trigger the plant secondary metabolism and increase phenolic compounds accumulation [25,26,27,28]. Moreover, wounding stress causes a significant effect on the phenylpropanoid pathway, thereby increasing the accumulation of phenolic compounds in plants [28]. In addition, wounding stress induces the expression of MYB transcription factors and regulate the flavonoid pathway genes expression [25]. In carrot, wounding stress increased the production and accumulation of caffeoylquinic acids [29,30]. In a recent study, secondary metabolites in the mechanically wounded leaves and roots of *Catharanthus roseus* seedlings were analyzed [31]. However, metabolic profile of the *C. roseus* callus was not reported. Several studies have examined the analytical chemistry, chromosomal biology, molecular biology, morphology, palynology, pharmacology, physiology, and transcriptomics of *Lycoris* [3,4,10,32,33,34,35,36,37,38]. However, GAL pathway (GP) gene expression and GAL content in wounded *Lycoris* have not been well studied. 

To this end, the present study evaluated the effects of wounding stress on the primary and secondary metabolism of *Lycoris radiata* callus based on GP gene expression and GAL accumulation. The results of this study provide comprehensive information on changes in the accumulation of GAL, which is of high commercial value, in *Lycoris* callus under wounding stress, and this information might be helpful for developing dietary supplements, pharmaceuticals, and functional foods.

## 2. Results 

### 2.1. Effects of Wounding Stress on GP Gene Expression

**Figure 2:**
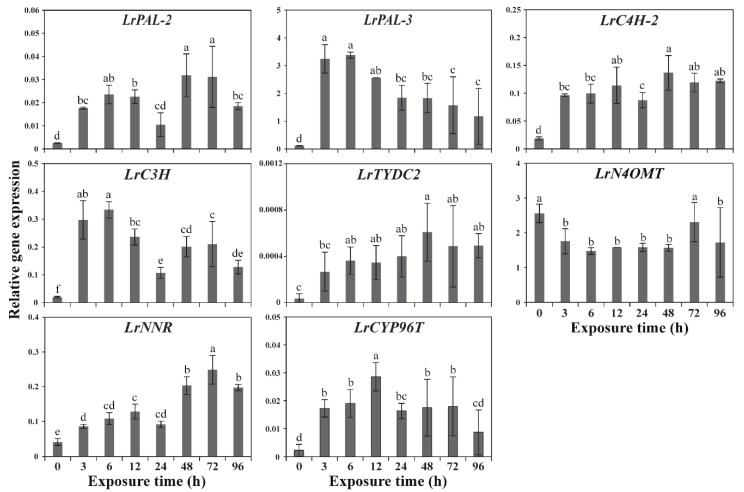
Relative gene expression levels of galantamine pathway genes at different time points following exposure to wounding stress. The housing keeping gene β-actin was used as internal control. Results are given as the mean of triplicates ± SD. Different letters indicate significant differences at *p* < 0.05.

The expression levels of eight GP genes (*LrPAL-2*, *LrPAL-3*, *LrC4H-2*, *LrC3H*, *LrTYDC2*, *LrN4OMT*, *LrNNR*, and *LrCYP96T*) in wounded *Lycoris radiata calli* were measured at various time points (0, 3, 6, 12, 24, 48, 72, and 96 h) following exposure using qRT-PCR. Of these, seven GP genes were significantly expressed at all the time points compared with the expression after 0 h of exposure (Figure 2). The expression levels of most GP genes were considerably increased with increasing exposure time. The expression level of *LrPAL-2* and *LrNNR* were considerably increased until 72 h and then gradually decreased after 96 h of exposure to wounding stress. However, there was no significant difference in *LrPAL-2* (24 h) and *LrNNR* (12 and 24 h) expression levels when compared to the control (0 h). The expression levels of *LrPAL-3* and *LrC3H* were the highest until 6 h and then gradually decrease in all other exposure time. This showed that PAL-2 does not have significant interaction with the GAL pathway. Moreover, the expression levels of *LrC4H-2* and *LrTYDC2* gradually induced with an increase up to 48 h and then slowly decreased after 72 and 96 h of exposure. In addition, the expression level of *LrCYP96T* considerably increased until 12 h and then decrease at subsequent time points, there was no significant difference in the expression level between the 0 and 96 h of exposure. However, there were no significant differences in *LrN4OMT* expression level between 0 and 72 h and subsequent time points following exposure to wounding stress.

### 2.2. Effects of Wounding Stress on GAL Content

**Figure 3:**
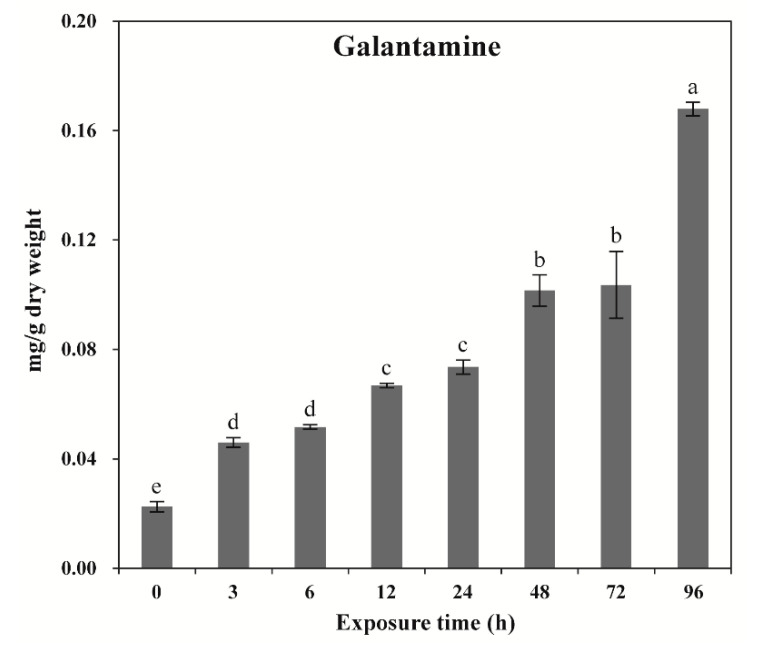
Galantamine content at different time points after exposure to wounding stress. Different letters indicate a significant difference at *p* < 0.05.

Exposure of *Lycoris radiata* calli to wounding stress induced a wide range of responses depending on the different exposure time. GAL content was significantly increased with increasing the exposure time. The highest GAL content was achieved at 96 h (0.17 mg·g^−1^ DW), followed by 72 and 48 h (0.10 mg·g^−1^ DW), 24 and 12 h (0.07 mg·g^−1^ DW), and 6 and 3 h (0.05 mg·g^−1^ DW) (Figure 3). The lowest GAL content was obtained after 0 h exposed callus (0.02 mg·g^−1^ DW) of exposure. Among the different times of exposure, the maximum fold was achieved after 96 h exposed callus (8.5-fold changes). 

### 2.3. Metabolic Profiling 

Calli exposed for 0 h that showed the lowest GAL content and calli exposed for 96 h that showed the highest GAL content were selected to investigate the effects of wounding stress on primary metabolites. Metabolic profiling using GC–TOFMS identified, 48 metabolites, including 12 organic acids, 22 amino acids, three sugar alcohols, seven carbohydrates, two carbohydrate derivatives, one amine, and 1 phenolic acid (Table S1). Further, after ninety-six-hour exposure to wounding stress, tyrosine, tryptophan, pyroglutamic acid, aspartic acid, valine, phenylalanine, leucine, lysine, glycine, and isoleucine contents significantly increased, while glutamine, glutamic acid, beta-alanine, methionine, cysteine, putrescine, alanine, and 4-aminobutyric acid contents decreased. Furthermore, the content of most carbohydrates, including glucose, galactose, fructose, mannose, arabinose, and glycerol, significantly increased, whereas those of sucrose and xylose decreased in 96 h of exposure. The content of ferulic acid, a well-known potent antioxidant, increased in calli exposed for 96 h.

**Figure 4:**
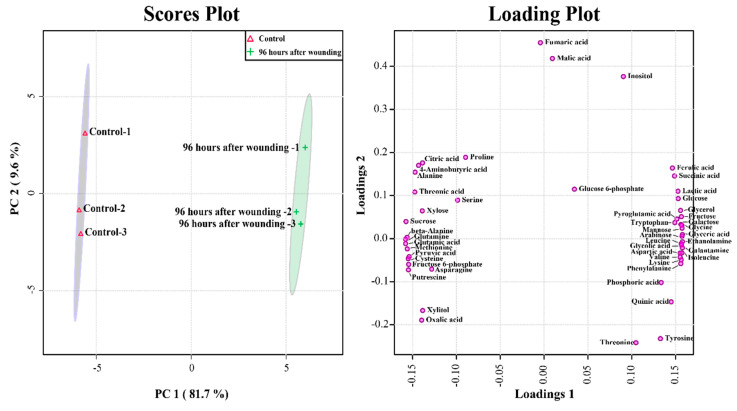
Score plots and metabolite differences between calli exposed for 0 and 96 h derived from a PCA model of GC-TOFMS results.

Principal component analysis (PCA) was used to determine differences in 49 metabolites between calli exposed for 0 and 96 h. The two highest-ranking components explained 91.1% of total variation (PC1: 81.3%; PC2: 9.8%). In particular, PC1 distinguished the calli exposed for 96 h from those exposed for 0 h, and the metabolites that contributed to this separation were mainly carbohydrates such as arabinose, mannose, fructose, galactose, and glucose (eigenvector values > 0.15), and amino acids, such as glycine, isoleucine, lysine, leucine, phenylalanine, aspartic acid, valine, tryptophan, pyroglutamic acid, and tyrosine (eigenvector values > 0.13). Furthermore, the eigenvector values of the glutamate family amino acids (glutamine, glutamic acid, and 4-aminobutyric acid) were lower than −0.14. Remarkably, eigenvector values of GAL and ferulic acid were 0.15785 and 0.14653, respectively, which are greater than the values reported for secondary metabolites, indicating that these metabolites explained the abovementioned increases in calli exposed for 96 h compared with the values in calli exposed for 0 h (Figure 4).

**Figure 5:**
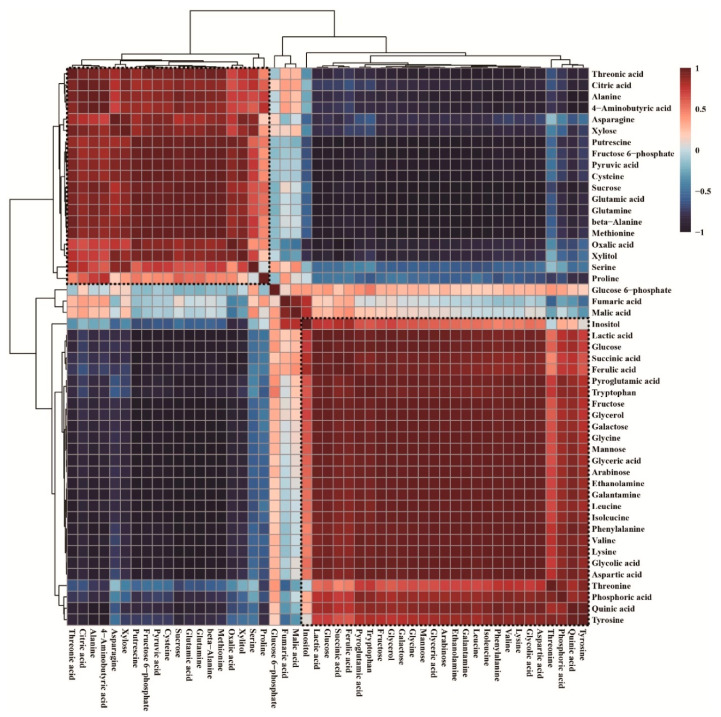
Correlation matrix-based hierarchical cluster analysis of results obtained from data of 49 metabolites between calli exposed for 0 and 96 h. Each square shows the Pearson’s correlation coefficient for a pair of metabolites, and the value for the correlation coefficient is displayed by the color difference, as shown on the color scale. Hierarchical clusters are characterized by a cluster tree.

GC-TOFMS data showed that the wounding stress increased contents of carbohydrates (arabinose, fructose, mannose, galactose, and glucose) and contents of amino acids (valine, leucine, isoleucine, glycine, aspartic acid, pyroglutamic acid, and phenylalanine). However, the levels of putrescine, glutamine, alanine, β-alanine, methionine, 4-aminobutyric acid, cysteine, glutamic acid, and asparagine decreased after wounding treatment. Furthermore, hierarchical cluster analysis (HCA) of Pearson’s correlation results obtained from data of 49 metabolites sets was used to investigate the associations among these 49 metabolites between calli exposed for 0 and 96 h exposed callus (Figure 5). HCA identified two major metabolite groups. One group had carbohydrates, such as arabinose, mannose, fructose, galactose, and glucose; amino acids such as glycine, isoleucine, lysine, leucine, phenylalanine, aspartic acid, valine, tyrosine, tryptophan, and pyroglutamic acid; GAL; ferulic acid; and organic acids. Glutamic acid and its derivatives, such as glutamine (*r* = 0.99877, *p* = 0.000002278) and 4-aminobutyric acid (*r* = 0.89758, *p* = 0.015198), were positively correlated. Similarly, amino acids of the erythrose 4-phosphate family (phenylalanine, tyrosine, and tryptophan) were positively correlated. Remarkably, GAL was strongly correlated with its precursors, phenylalanine (*r* = 0.99271, *p* = 0.000079595) and tyrosine (*r* = 0.86316, *p* = 0.026808), in that order. Carbohydrates function as energy sources for the production of secondary metabolites. In this study, the strong correlations were observed between GAL and carbohydrates, including glycerol (*r* = 0.97718, *p* = 0.00077507), inositol (*r* = 0.53438, *p* = 0.27473), glucose (*r* = 0.95686, *p* = 0.0027511), fructose (*r* = 0.98507, *p* = 0.00033272), galactose (*r* = 0.98412, *p* = 0.00037622), mannose (*r* = 0.99237, *p* = 0.000087), and arabinose (*r* = 0.99409, *p* = 0.0000522). Similarly, ferulic acid was strongly correlated with its precursor phenylalanine (*r* = 0.88154, *p* = 0.000079595) and carbohydrates (glycerol, inositol, glucose, fructose, galactose, mannose, and arabinose) with *r* > 0.8 and *p* < 0.05. 

## 3. Discussion

qRT-PCR revealed that eight of the seven GP genes (*LrPAL-2*, *LrPAL-3*, *LrC4H-2*, *LrC3H*, *LrTYDC2*, *LrNNR*, and *LrCYP96T*) analyzed were differentially expressed due to wounding conditions. Several previous studies have reported that increased expression levels of all these genes enhanced GAL accumulation in Amaryllidaceae plants, such as *Narcissus* sp. *aff*. *pseudonarcissus* and *Lycoris radiata* [4,24]. In addition, significant expression of *PAL*, *C4H*, *4CL*, *CCoAOMT*, *CAD*, and *CCR* increased phenolic compound accumulation in various plants, such as *Arabidopsis thaliana* [39,40], *Daucus carota* [41], *Lactuca sativa* L., var. Longifolia [42], and *Populus trichocarpa* [43]. In wounded *Lycoris radiata* calli, the GP genes (*LrPAL-2*, *LrPAL-3*, *LrC4H-2*, and *LrC3H*) were significantly expressed, suggesting that the *PAL* and *C4H* are the two essential enzymes in the phenylpropanoid pathway, which is associated with the production of numerous important phenolic compounds in plants, such as anthocyanin, flavonoid, lignin, phenolic acid, and suberin, etc. [44]. Moreover, increase in *LrPAL-3* and *LrC3H* expression might be an intermediate step in the synthesis of various phenolic compounds in wounded *Lycoris radiata* calli. However, further studies are warranted in the future to confirm this hypothesis. Furthermore, an increase in *LrNNR* and *LrCYP96T* expression and GAL accumulation in wounded *Lycoris radiata* calli indicate that the late-expressing GP genes especially *LrNNR* and *LrCYP96T* play pivotal roles in GAL biosynthesis compared with the early-expressing GP genes. This trend is consistent with a previous report that the expression of late GP genes was increased in *Lycoris radiata*, which further increased GAL content [4].

Previous studies reported that *Narcissus* sp. bulbs showed the highest accumulation of GAL content due to the highest expression of NpN4OMT [24]. A similar result was obtained in bulbs of *Lycoris radiata* the expression level of *LrNNR* and *LrN4OMT* was highly expressed which leads to a significant accumulation of GAL content. In addition, they had reported that the expression of late expressed genes (*NNR* and *N4OMT*) is more important than the early expressed genes (*PAL* and *C4H*) in the GP [4]. This leads to the highest accumulation of GAL content in the *Lycoris radiata* bulb. However, in this study, the *LrNNR* is significantly increased with increasing the exposure time, whereas *LrN4OMT* genes were downregulated in all the exposure time. Moreover, in the wounded *Lycoris radiata* callus the GAL content (0.17 mg·g^−1^ DW) was lower when compared to the root (0.53 mg·g^−1^ DW), bulb (0.75 mg·g^−1^ DW), and leaf (0.27 mg·g^−1^ DW) of *Lycoris radiata* [4]. Taken together, these results it is suggested that NNR and N4OMT act together in the GP for significant accumulation of GAL content in plants. From this study result, it is shown that N4OMT is more important than the NNR for the highest accumulation of GAL content.

In the present study, we found that GAL accumulation was significantly increased with increasing exposure time. Some of the studies had reported that wounding stress significantly increases the contents of benzylisoquinoline alkaloids, shikimic acid, primary or secondary metabolites [31,45,46]. Specifically, integrated transcriptomic and metabolomic analysis of benzylisoquinoline alkaloids in wounded *Papaver somniferum* revealed significant increases in the narcotine and papaverine accumulation [45]. Moreover, wounded carrot tissues showed enhanced shikimic acid accumulation [46]. In addition, wounding stress stimulated the production of chlorogenic acid (CHA), 3,5-dicaffeoylquinic acid, and 4,5-dicafeoylquinic acid in carrot [46]. Similarly, mechanical wounding of *Catharanthus roseus* leaves and roots considerably increased primary and secondary metabolite contents in the wounded tissue [31].

Phenylalanine and tyrosine are the initial precursors of GAL and ferulic acid. The present study showed the strongest positive correlation between these amino acids as well as between these secondary metabolites, suggesting that the wounding stress increased endogenous phenylalanine and tyrosine levels, thus enhancing GAL and ferulic acid biosynthesis. These findings are consistent with previous reports that increase in endogenous phenylalanine level due to cessation of cell division likely enhanced the expression of *PAL* and chalcone synthase, ultimately increasing anthocyanin production in *Vitis* cells [47]. Exogenous phenylalanine application markedly affected the production of free phenolic acids (e.g., gallic acid, CHA, syringic acid, protocatechuic acid, and vanillic acid) in leaves of *Pisum sativum* through enhancing *PAL* activity [48]. Furthermore, exogenous application of phenylalanine and tyrosine increased the contents of total catechin, total proanthocyanidin, and total phenolics in *Triticum aestivum* L [49] as well as enhanced the accumulation of phenylethanoid glycosides in cell cultures of *Cistanche deserticola* [50]. Dietary intake of phenylalanine increased the levels of *p*-coumaric acid—a precursor of ferulic acid—in cell cultures of *Larrea divaricata* Cav. [51]. Unfortunately, there are no previous studies on the effects of wounding stress on GAL biosynthesis. 

Carbohydrates function as energy sources for the production of secondary metabolites. This study showed a strong positive correlation between secondary metabolites (GAL and ferulic acid) and carbohydrates, including glycerol, inositol, glucose, fructose, galactose, mannose, and arabinose. These results are in agreement with previous studies reporting that the abundance of endogenous carbohydrates is likely to be correlated with the abundance of secondary metabolites. For example, Park et al. [52] reported that the large pools of carbohydrates (sucrose, raffinose, maltose, xylose, mannose, fructose, glucose, mannitol, myo-inositol, and glycerol) were correlated with large pools of phenolics and glucosinolates in Chinese cabbage. Similarly, the strong positive correlation between phenolic compounds and carbohydrates in flowers of *Magnolia Denudata* Desr. and *Magnolia Liliiflora* Desr. was observed [53].

## 4. Materials and Methods

### 4.1. Callus Induction

*Lycoris radiata* was grown in a greenhouse at the Department of Crop Science, Chungnam National University, Daejeon, Korea. *Lycoris radiata* bulbs were harvested and washed first with running tap water and then with 70% ethanol for 1 min. In a laminar airflow chamber, the bulb was soaked in 4% NaOCl for 12 min, washed 4–5 times with sterile deionized water, and dried with a sterile tissue paper. The meristems of the dried sterile bulbs were cut into sections measuring 0.5 cm × 0.5 cm, which were transferred to a Petri plate containing agar-solidified callus induction medium. The medium contained Murashige and Skoog salts and vitamins, 30 g·L^−1^ sucrose, 2 mg·L^−1^ 2,4-D, 0.5 mg·L^−1^ TDZ, and 2 mL·L^−1^ plant preservative mixture and was solidified with 0.8% (w/v) phytagar. pH of the media was adjusted to 5.8 before adding agar, and the medium was then sterilized by autoclaving at 121 °C for 20 min. Cultures were maintained in a growth chamber in the dark at 25 ± 1 °C. For callus induction from the meristem, the explants were subcultured onto fresh callus induction medium every month for 6 months. After 6 months, compact calli were obtained.

### 4.2. Wounding Treatment

For wounding experiments, callus cultures were collected in sterile Petri plate, and the calli were wounded by repeatedly cutting into the cellular mass with a sterile scalpel blade. The 5 g wounded calli were placed on the callus induction medium and maintained in a growth chamber in the dark at 25 ± 1 °C. The calli induced on one medium is one replicate and three replicates were prepared for each time point. The wounded calli were harvested at different time points (0, 3, 6, 12, 24, 48, 72, and 96 h after wounding) and stored at −80 °C for RNA extraction and metabolic compound analysis. All experiment samples were prepared and analyzed in triplicates.

### 4.3. RNA Extraction, cDNA Synthesis, and qRT PCR

Total RNA was extracted from wounded *Lycoris radiata* calli by using Easy BLUE Total RNA Kit (iNtRON, Seongnam, Korea). The quality and quantity of total RNA were analyzed by using 1.2% denaturing agarose gel and NanoVue Plus spectrophotometer (GE Healthcare, Chicago, IL, USA), respectively. Total RNA (1 ng) from each sample was used for first-strand cDNA synthesize using the ReverTra Ace kit (Toyobo Co., Ltd., Osaka, Japan). Then, the first-strand cDNA templates were diluted 2-fold with nuclease-free water for qRT-PCR. All qRT-PCR runs were performed with BioFACT™ 2X Real-Time PCR Master Mix kit with SFCgreen^®^ I (BioFACT, Daejeon, Korea) on a CFX96TM Real-Time PCR Detection System (Bio-Rad, Hercules, CA, USA). The qRT-PCR conditions were as follows; 15 min at 95 °C (pre-denaturation), followed by 40 cycles of 15 s at 95 °C (denaturation), 30 s at 55 °C (annealing), and 20 s at 72 °C (extension). All the reaction was done in three biological replicates. To quantify the expression levels of GP genes in wounded *Lycoris radiata* calli, the housing keeping gene β-actin was used as the internal control. The primer used for qRT-PCR are shown in Table S2. Cq values of β-actin at different time points after exposure to wounding stress (Table S3). 

### 4.4. High-Performance Liquid Chromatography (HPLC)

GAL extraction and analysis were done according to the protocol described in a previous study Park et al. [4] with slight modification. For extraction, fine powdered samples (100 mg) of wounded *Lycoris radiata* calli collected at different time points after exposure were used and add 2 mL of 0.1% trifluoroacetic acid was added. Then the samples were sonicated for 30 min, and stored overnight at 4 °C. The next day the samples were taken and centrifuged for 10 min at 13,000 rpm, and the supernatant was filter-sterilized through a 0.45 µm Acrodisc syringe filter (Pall Corp., Port Washington, NY, USA). GAL was analyzed using the NS-4000 HPLC system with a C_18_ column (250 mm × 4.6 mm, 5 μm) at 30 °C and detected with the NS-6000 auto-sampler and a UV–Vis detector (Futecs Co., Daejeon, Korea) at 285 nm. The mobile phase comprised 50mM ammonium formate aqueous buffer (solvent A) and acetonitrile (solvent B), and the flow rate was 1 mL·min^−1^. The following gradient program (38 min in total) was used: 2% solvent B, 0–15 min; 2–65% solvent B, 30–31 min, 65–100% solvent B, 31–35 min; 100% solvent B, 35–36 min, 100–2% solvent B; and 2% solvent B, 36–38 min. For each run, 20 µL of each sample was injected. GAL content was estimated according to the protocol described in a previous study Park et al. [4]. All the analyses were performed in triplicate.

### 4.5. Gas Chromatography–Time-of-Flight Mass Spectrometry (GC–TOFMS)

Hydrophilic metabolites were extracted according to the protocol described in a previous study Park et al. [4] with slight modification. In brief, wounded *Lycoris radiata* callus samples (10 mg) collected at different time points after exposure were used and 1 mL of water/CHCl_3_/MeOH (1:1:2.5, *v*/*v*/*v*) followed by 60 µL of 0.2 g L^−1^ adonitol (internal standard) was added. The extraction was performed in a compact thermomixer at a mixing frequency of 1200 rpm at 37 °C for 30 min. The polar phase of the samples was separated by centrifugation at 10,000 rpm for 5 min, 800 µL of the polar phase was transferred to a sterile Eppendorf tube, to which 400 µL of deionized water. Then this mixture was centrifuged at 10,000 rpm for 5 min to separate the methanol–water phase containing the polar metabolites, and it was evaporated with a centrifugal concentrator (CVE-2000, Daejeon, Korea) for 3 h. The residues were lyophilized for 15 h by using freeze dryer. The lyophilized materials were furtherly processed through two steps: methoxide derivatization and trimethylsilyl etherification. Methoxyamine hydrochloride/pyridine (20 g·L^−1^, 80 μL) was added to a vial and it was shaken for 90 min at 30 °C. After centrifugation, 80 μL of *N*-methyl-*N*-(trimethylsilyl) trifluoroacetamide was added to the vial, and the mixture was heated at 37 °C for 30 min. The resulting products were analyzed using Agilent 7890 GC system (Agilent, Atlanta, GA, USA). The operating condition, flow rate, and gradient program followed the protocol described previously by Park et al. [4]. The metabolites were identified using an in-house library and quantified based on the ratio of the peak areas of metabolites relative to the peak area of the internal standard.

### 4.6. Statistical Analysis

Data were analyzed by Duncan’s multiple range test, with a significance level of *p* < 0.05, using SAS 9.4 (SAS Institute, Inc., Cary, NC, USA). Duncan’s multiple range test, a method to make comparisons between groups of means, has been widely used in the plant science field since its procedure is simple and easy to perform it. Therefore, the test was considered appropriate in this study. Principal component analysis (PCA) and Pearson correlation analysis for 49 metabolites, detected in calli exposed for 0 h and calli exposed for 96 h using HPLC and GC-TOFMS, were carried out using the MetaboAnalyst 4.0 (http://www.metaboanalyst.ca/) with auto-scaling. Correlation matrix and hierarchical clustering were created using the MetaboAnalyst 4.0. The hierarchical clustering based on the Euclidean distance coefficient and Ward’s linkage method was carried out and the color key is based on Pearson correlation coefficients.

## 5. Conclusions

In conclusion, it was confirmed that the wounding increased GAL accumulation by enhancing its biosynthesis through the phenylpropanoid pathway in *Lycoris radiata* calli. The qRT-PCR analysis showed that seven out of eight upregulated genes were significantly upregulated in *Lycoris radiata* calli. In particular, wounding gradually upregulated the expression of *LrPAL-2*, *LrPAL-3*, *LrC4H-2*, *LrC3H*, *LrTYDC2*, *LrNNR*, and *LrCYP96T* in *Lycoris radiata* calli, thereby increasing GAL accumulation was gradually increased from 3 h to 96 h of exposure to wounding stress, and this might be due to the enzymatic reaction of the seven genes. In addition, GC-TOFMS result showed a strong positive correlation between galantamine and initial precursors, such as phenylalanine and tyrosine, and a strong correlation between galantamine and carbohydrates, as energy sources, was observed. Our results suggest that secondary metabolite contents in cell cultures of *Lycoris radiata* can be increased by exposing calli to wounding stress.

## Supplementary Materials

The following are available online at https://www.mdpi.com/2223-7747/9/11/1616/s1, Table S1: Metabolite peak height differences using GS-TOFMS (ratio/g). Table S2. Primers used for qRT-PCR. Table S3: Cq values of β-actin at different time points after exposure to wounding stress.
