# Supplementary material for: Metabolic Profiling of Primary Metabolites and Galantamine Biosynthesis in Wounded Lycoris radiata Callus
Source: Plants (Basel). 2020 Nov 20;9(11):1616. doi: 10.3390/plants9111616 (PMC7699913; doi:10.3390/plants9111616)
Supplement: Supplementary file 1 [file plants-09-01616-s001.pdf]

Table 1. Metabolite peak height differences using GS-TOFMS (ratio/g).

Name	W0hr-1	W0hr-2	W0hr-3	W96hr-1	W96hr-2	W96hr-3
Pyruvic acid	0.00216	0.002886	0.002356	0.00066	0.000649	0.000766
Lactic acid	0.012455	0.011048	0.010408	0.01885	0.016566	0.017259
Alanine	0.933805	0.915716	0.896552	0.835702	0.794857	0.773223
Oxalic acid	0.008364	0.009739	0.010701	0.006775	0.00721	0.007268
Glycolic acid	0.002753	0.003617	0.002562	0.011146	0.010427	0.01163
Valine	0.188209	0.200164	0.194796	0.239082	0.238658	0.235109
Serine	0.096551	0.11742	0.078806	0.083308	0.069537	0.082119
Ethanolamine	0.032663	0.03189	0.030398	0.106935	0.106139	0.108875
Glycerol	0.074695	0.070358	0.068576	0.12962	0.117891	0.115131
Phosphoric acid	0.199746	0.231579	0.206029	0.243627	0.249002	0.242801
Leucine	0.068363	0.070892	0.078311	0.18038	0.183303	0.164247
Isoleucine	0.064717	0.0715	0.073997	0.171003	0.176892	0.170331
Proline	0.710216	0.680277	0.712137	0.69433	0.685035	0.636493
Glycine	0.163754	0.16382	0.158255	0.246608	0.23524	0.233844
Succinic acid	0.075166	0.040759	0.027703	0.139552	0.122253	0.123297
Glyceric acid	0.009063	0.006618	0.005028	0.05769	0.055769	0.057489
Fumaric acid	0.020976	0.008627	0.005363	0.016491	0.009084	0.007829
Threonine	0.062923	0.067079	0.065125	0.0669	0.067224	0.067783
β-Alanine	0.116096	0.120929	0.112747	0.082823	0.083155	0.08642
Malic acid	0.137701	0.133091	0.125691	0.136337	0.130017	0.131132
Aspartic acid	0.041984	0.04453	0.042194	0.052681	0.052051	0.053174
Methionine	0.011158	0.012619	0.011114	0.004851	0.005158	0.005741
Pyroglutamic acid	0.378476	0.406864	0.360169	0.513026	0.485823	0.48253
4-Aminobutyric acid	0.538502	0.519999	0.518896	0.480645	0.445792	0.429393
Threonic acid	0.036618	0.036238	0.030303	0.023768	0.022776	0.02359
Cysteine	0.004133	0.005336	0.004486	0.001047	0.001185	0.001439
Glutamic acid	0.269128	0.281424	0.276492	0.155493	0.157317	0.158897
Phenylalanine	0.02951	0.035755	0.032317	0.064387	0.06592	0.06976
Xylose	0.004878	0.005686	0.004392	0.003758	0.003262	0.003444
Arabinose	0.001462	0.00158	0.001271	0.008892	0.00779	0.008777
Asparagine	0.178277	0.203861	0.180892	0.165873	0.163044	0.167181
Xylitol	0.010399	0.012666	0.011861	0.008802	0.008889	0.009681
Putrescine	0.046106	0.051621	0.053957	0.028347	0.027848	0.030036
Glutamine	0.349112	0.360395	0.349573	0.175086	0.186048	0.17844
Citric acid	0.064898	0.064915	0.058911	0.054597	0.043861	0.045394
Quinic acid	0.011172	0.011887	0.011806	0.013108	0.013172	0.014059
Fructose	0.527528	0.503692	0.469275	1.003951	0.915207	0.916939
Mannose	0.007703	0.007421	0.00726	0.022797	0.020546	0.020788

Galactose	0.004913	0.004924	0.004125	0.029795	0.027695	0.023782
Glucose	0.098239	0.091757	0.08363	0.187432	0.1551	0.16103
Lysine	0.054755	0.061305	0.059722	0.091551	0.090309	0.09228
Tyrosine	0.055668	0.071578	0.068721	0.078885	0.085539	0.092274
Inositol	0.121518	0.113651	0.11082	0.126408	0.118591	0.117818
Ferulic acid	0.001142	0.000928	0.000717	0.001827	0.001526	0.001579
Tryptophan	0.004335	0.007143	0.003418	0.016424	0.01204	0.014611
Fructose 6-phosphate	0.001158	0.001429	0.001237	0.000376	0.000452	0.000554
Glucose 6-phosphate	0.001369	0.001542	0.001411	0.001659	0.001331	0.001468
Sucrose	0.318206	0.295372	0.287066	0.016765	0.008682	0.006588

Table 2. Primers used for qRT-PCR.

Genes	Forward primer (5' to 3')	Reverse primer (5' to 3')
LrPAL-2	GGGCTACCTACAAATCTCTC	CATCTTGGTTGTGTTGGTTCC
LrPAL-3	GCAAACACTTCCATCTTCCA	CGTCTTCTCTCACAAACCGA
LrC4H-2	AGTCGCTTCTCGCCGTATTC	AGGTTGCGGTGGTTGAG
LrC3H	GACCACCCTCAACATCGT	GCAGACCTTCCTCACCTT
LrTYDC2	TTTGCCCAGAATACCGACA	AGAGCAGGGAGCAATCAAAG
LrNO4MT	GCTGAGGGAGGTGACTGA	ATTGCCGTTATCTTTCCATC
LrNNR	CGTTTGTGGAGGATAAGGA	AGTGATGTAGGAGACAGATG
LrCYP96T	CGATGCCGTGTCTTTCTAC	GGATTGCGTGTTCTCTGC
LrActin	AGGAATGGGTCAAAAGGATG	TTGGCTTTCGGGTTCAGAG

Table 3. Cq values of β-actin at different time points after exposure to wounding stress.

Time (hour)	Cq values
0	25.48 ± 0.11
3	25.27 ± 0.21
6	25.63 ± 0.12
12	25.86 ± 0.03
24	25.49 ± 0.06
48	25.71 ± 0.13
72	25.96 ± 0.32
96	25.31 ± 0.08
